# Aerobic capacity of professional soccer players before and after COVID-19 infection

**DOI:** 10.1038/s41598-022-16031-7

**Published:** 2022-07-13

**Authors:** Koulla Parpa, Marcos Michaelides

**Affiliations:** University of Central Lancashire, Cyprus Campus, University Avenue 12-14, 7080 Pyla, Cyprus

**Keywords:** Physiology, Diseases

## Abstract

This investigation aimed to assess the aerobic capacity of professional soccer players pre-and post-COVID-19 infection. Twenty-one division-1 elite soccer players (age 24.24 ± 5.75 years, height 178.21 ± 5.44 cm, weight 74.12 ± 5.21 kg) participated in this study. This observational study compared the same players' aerobic capacity pre-, and 60-days post COVID-19 recovery. The statistical analysis demonstrated that the infected players had significantly lower VO_2max_ values [t_(20)_ = 5.17, p < 0.01, d = 0.613 (medium effect)], and significantly lower VO_2_ values at respiratory compensation point (RC) [t_(20)_ = 2.97, p < 0.05, d = 0.39 (small effect)] after recovery. Furthermore, results indicated a significantly lower running time (RT) on the treadmill [t_(20)_ = 4.84, p < 0.01, d = 0.46 (small effect)] when compared to the results that were obtained before they got infected. In addition, velocity at VO_2max_ (_V_VO_2max_) was significantly lower [t_(20)_ = 2.34, p < 0.05, d = 0.41 (small effect)] and the heart rate values at ventilatory threshold (VT) [t_(20)_ = −2.79, p < 0.01, d = 0.55 (medium effect)] and RC [t_(20)_ = −3.72, p < 0.01, d = 0.52 (medium effect)] were significantly higher post-recovery. The aforementioned findings indicate that post COVID-19 soccer players may not reach full recovery at two months. Therefore, our results highlight that further adaptations and improvements are needed with regard to aerobic capacity before soccer players return to professional games.

## Introduction

Coronavirus, a highly infectious disease currently termed severe acute respiratory syndrome coronavirus 2 (SARS-CoV-2), is generally accompanied by mild to moderate manifestations^1^. However, a small proportion of patients develop a severe respiratory illness that may lead to death in some cases^1^. The lungs are the organs primarily affected by COVID-19 as the virus accesses host cells via the enzyme ACE2 (angiotensin-converting enzyme 2), which is most abundant in alveolar (Type II) cells of the lungs^2^. Once the spike glycoproteins of SARS-CoV-2 connect to ACE2, the virus enters the host cell^2^. Thus, even though the lungs are the most affected organs by COVID-19, the virus can spread to other organs and infect ACE2-expressing cells at local sites, causing multi-organ problems^2^. Considering that ACE2 receptors are highly expressed in the heart, that may explain the acute myocardial injuries that have been noted as complications in patients infected with SARS-CoV-2^2^. It is worth noting that the exact pathophysiology of COVID-19 remains unclear, and cardiac injury is reported to result from direct or indirect mechanisms^3^.

The infection rate among professional soccer players is consistent with that of the general population^4^, and most of the time, COVID-19 positive athletes are asymptomatic^6^. However, they may experience mild to moderate symptoms such as fever, cough, loss of taste or smell, headache, aches, muscle pain, sore throat, and tiredness^4–6^. Less common and more severe symptoms include shortness of breath, pain and pressure in the chest, or pneumonia^5^. It has been recommended that symptomatic athletes with moderate manifestations should rest from exercise during the symptomatic phase and for at least 14 days after the complete resolution of symptoms, while asymptomatic patients should not resume physical activity for at least 14 days after diagnosis^5^. Conclusively, COVID-19 positive professional soccer players find themselves in a unique situation in which they are not only obliged to be self-isolated but also, to abstain from any form of physical activity for at least 14 days after diagnosis.

Positive COVID-19 soccer players could potentially have even higher psychological and physiological strain than that reported during quarantine periods^7, 8^. Notably, the lockdown alone has been indicated to evoke a negative effect on mental wellbeing^9^ and emotional status, with a great proportion of individuals experiencing psychosocial and emotional disorders^7^. Not surprisingly, an increase in sedentary behavior during leisure time was associated with poorer physical health, mental health, and subjective vitality^10^. In addition to the psychological distress, a multilingual online survey of 5056 participants affirms that COVID-19 confinement led to impaired sleep quality that was related to sleep disturbances, daytime dysfunction, the use of sleep medications, and sleep latency [^8^]. Additionally, although home-based training during lockdown effectively improved aerobic fitness^11, 12^, athletes' competitive power levels were not maintained^11^. Research affirms that long term detraining (more than four weeks) causes a significant reduction in aerobic capacity, resulting in lower stroke volume and cardiac output, despite increased heart rates^13^. Unlike reduced physical fitness after a prolonged period of detraining in elite athletes^13, 15^, the effects of short-term detraining (~ 2 weeks) on fitness are controversial. Some studies reported that even a short period of inactivity might have a significant detraining effect^14^. In particular, 2-weeks of inactivity caused a marked reduction in aerobic capacity and repeated sprint ability in semi-professional soccer players^14^. On the contrary, it was indicated that short-term detraining after a competitive season improved levels of strength and cardiorespiratory fitness in Australian soccer players^16^.

In soccer, physical fitness is heavily dependent upon aerobic capacity, as it is well documented that during professional soccer games, players cover total distances of 9–14 km^17^. Furthermore, although high-intensity demands are critical during a soccer game^24^, the aerobic energy system's predominance is evident during low- to moderate intensity running demands. Thus, aerobic capacity increases the distance covered during a game, the number of sprints, and interactions with the ball^26^. Notably, this parameter becomes increasingly important in soccer players after COVID-19 infection.

It is unknown whether soccer players who have been infected and recovered have residual cardiorespiratory complications, as there are no clinical data to indicate that. However, earlier reports in SARS patients (2003 outbreak) suggest the possibility of cardiorespiratory impairments in athletes even 24 months after SARS onset^35^. Therefore, the aim of this study was to examine the aerobic capacity of professional soccer players before and after COVID-19 infection. It was hypothesized that professional soccer players' aerobic capacity would not significantly differ two months post-COVID infection compared to pre-infection.

## Materials and methods

### Subjects

This observational study compared the same players' aerobic capacity pre- and 60-days post-COVID-19 infection. The soccer players of three teams were tested at the end of April (pre-testing) as part of their teams' assessment before the playoffs (Table 1). Players who underwent the initial testing and have tested positive for COVID-19 during the following months were recruited for this study. Those that reported two or more mild to moderate symptoms of body discomfort were included in the study. Asymptomatic players and those who have tested positive after being vaccinated were excluded from the study. In addition, infected goalkeepers were excluded from the study as they did not follow the same re-training and adaptation program as the in-field players after they recovered. Conclusively, a total of twenty-one division 1 soccer players (age: 24.24 ± 5.75, height 178.21 ± 5.44 cm, weight 74.12 ± 5.21 kg) who met the inclusion criteria were tested 60-days post-COVID infection.

**Table 1:** Testing timeline.

2021	Jan	Feb		March	April		May	June		July	August	
Second lockdown	31 days											
Official games		1–15 days	16–28 days	31 days	1–15 days							
Pre-testing						16–30 days						
COVID-19 positive recruitment							31 days	1–15 days				
2-weeks no training									16–30 days			
Adaptation program										31 days	1–15 days	
Post-testing												16–30 days

As reported by the team's staff and medical group, prior to the resumption of vigorous training, the players had a minimum of two consecutive negative PCR tests,a confirmed IgM negative test and a specific health evaluation that included a cardiology assessment, under the condition that at least 14 days have passed since the positive test. Furthermore, before the post-testing, the players followed a 2-week re-training program based on the safe return to sport activities guidelines^18^. In addition, they followed a 10-days specific adaptation program and a 20-days game adaptation program based on the guidelines^18^ (Table 2).

**Table 2:** Phases: return to training and games following COVID-19 infection.

Phase	Exercises	Sessions	Duration
Re-training	Individual football specific exercises	1 per day (max 45–60 min)	14 days
	Focus on aerobic conditioning without strength and explosive power training		
	All exercises performed at low to moderate intensity		
	Attention to load distribution		
Specific adaptation	Football specific training exercises in a group	1 per day (max 75 min)	10 days
	Gradual increase of session training intensity with appropriate recovery time		
	Position specific training activities		
Game adaptation	Introduction to high-speed movements such as sprinting, kicking and changes of direction	1 per day (max 75–90 min)	20 days
	Introduction to match play drills		
	Friendly games with attention to individual minutes played		
	Progressive increase in participation minutes during friendly games		
	Application of specific recovery protocols post games and the day after		

### Procedures

All the participants had medical clearance and a negative COVID-19 polymerase chain reaction (PCR) test within 48–72 h before the testing despite recovering from COVID. Players were advised to abstain from any activity the day before testing, and measurements were obtained at approximately the same time of the day for both the pre-and post-testing. Players' participation in this study was completely voluntary, and each player was briefed on the procedures before they signed an institutionally approved written informed consent form. The study was carried out in accordance with the Declaration of Helsinki and was approved by the University's ethics committee board and the National Committee on Bioethics. The researchers were required to wear a face mask, face shield, gown, and gloves during data collection and adhered to the guidelines for patients and testing personnel during aerosolizing procedures as indicated by the Centers for Disease Control and Prevention^19^.

### Anthropometric measurements

Anthropometric measurements were recorded using a wall stadiometer (Leicester; Tanita, Tokyo, Japan) to measure the players' stature and a leg-to-leg bioelectrical impedance analyzer (BC418MA; Tanita) to measure body composition.

### Cardiopulmonary exercise testing

Before the cardiopulmonary testing, players performed a 5-min self-paced warm-up on a mechanically braked cycle ergometer (Monark 894 E Peak Bike, Weight Ergometer, Vansbro, Sweden). After that, the players completed an incremental maximal cardiopulmonary exercise testing (CPET) until they reached exhaustion on a treadmill (h/p/Cosmos Quasar med, H-P-Cosmos Sports & Medical GmbH, Nussdorf-Traunstein, Germany). A breath-by-breath analysis was performed on the Cosmed Quark CPET (Rome, Italy) system. Laboratory conditions were kept constant, with the temperature at 22 ± 1 °C and relative humidity at 50%. The players were tested utilizing the modified Heck incremental maximal protocol, which was previously validated for its reliability in testing soccer players^20^. The protocol was composed of a warm-up, exercise and a recovery phase. The inclination was kept constant at 3% for the warm-up and exercise phases. The warm-up phase speed started at 4.8 km/h and increased by 1.2 km/h every 1 min. The exercise phase speed started at 8.2 km/h and increased by 1.2 km/h every 2 min to exhaustion. The recovery phase speed was reduced to 4.8 km/h and remained constant for 3 min with no inclination. The test came to an end when the participant reached volitional fatigue or when there was no variation among the VO2 levels while the workload increased. Breath-by-breath VO_2_ data were time-averaged across ten seconds intervals. Only complete breaths were included in each discrete block of time-averaged VO2 data. The highest averaged VO_2_ value was regarded as VO_2max_. The total running time (RT) on the treadmill was recorded in minutes and included in the data analysis.

### Determination of ventilatory threshold and respiratory compensation point

The ventilatory threshold (VT) and the respiratory compensation point (RC) were determined using different criteria. VT is commonly described as the point at which pulmonary ventilation and carbon dioxide (CO_2_) output begin to increase exponentially^21^, while RC represents the point at which lactate is rapidly increasing with intensity and is associated with hyperventilation. The VT was determined through the V-Slope method, the point at which the increase in the rate of elimination of carbon dioxide ($\dot{\rm V}$ CO_2_) is greater than the increase in $\dot{\rm V}$ O_2_. The VT point was verified at the nadir of the VE/$\dot{\rm V}$ O_2_ curve. The RC point was determined at the nadir of the VE/$\dot{\rm V}$ CO_2_ curve^22, 23^. The plots used to determine the thresholds utilized filtered breath-by-breath values (averaged into 10-s bins). Two experienced exercise physiologists completed all the assessments of VT and RC. The original assessments were reevaluated if there were conflicting results, and a consensus was reached.

### Statistical analyses

SPSS 26.0 for Windows (SPSS Inc., Chicago) was used for analyzing the results. Normality and homogeneity of variances were examined and verified using the Shapiro–Wilk and the Brown and Forsythe tests, respectively. The mean and SD were calculated for all parameters. Paired t-tests were used to identify the differences between the pre-and post-measurements in aerobic capacity. Cohen's d was calculated to determine the effect size. Effect sizes were interpreted as follows: small (0.2–0.4), medium (0.5–0.7), and large (0.8–1.4)^24^. For all statistical analyses, significance was accepted at p < 0.05.

## Results

**Figure 1:**
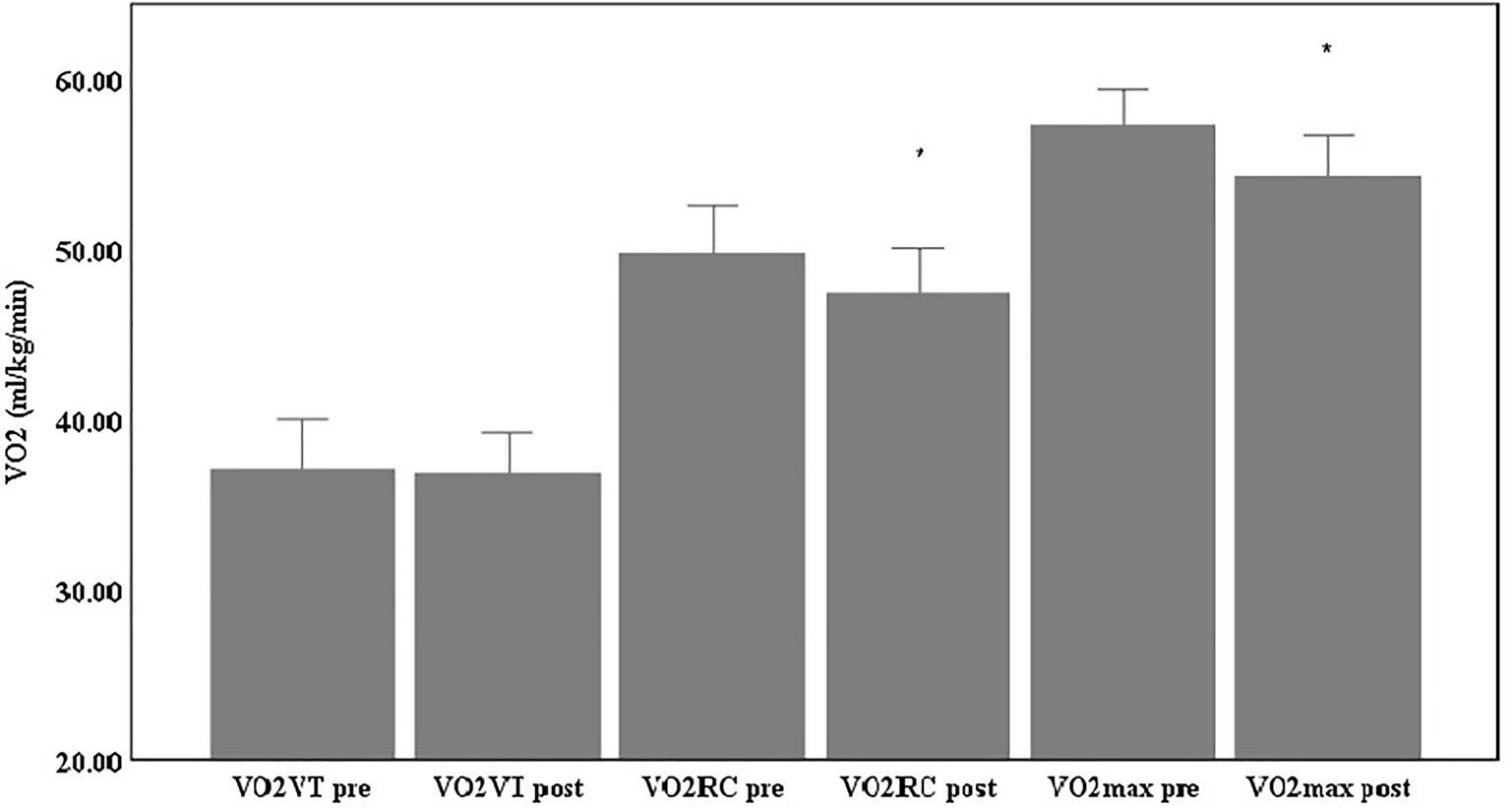
VO_2max_, VO_2VT_ and VO_2RC_ pre- and post-COVID-19.

The anthropometric and body composition measurements are presented in Table 3. Results demonstrated that the infected players had significantly lower VO_2max_ values [t_(20)_ = 5.17, p < 0.01, d = 0.613 (medium effect)], and significantly lower VO_2_ values at RC (VO_2RC_) [t_(20)_ = 2.97, p < 0.05, d = 0.39 (small effect)] 60 days after recovery (Table 4, Fig. 1). At the same time no significant difference was demonstrated in the VO_2_ values at VT (VO_2VT_) following COVID-19 recovery. Furthermore, results indicated a significantly lower RT on the treadmill [t_(20)_ = 4.84, p < 0.01, d = 0.46 (small effect)], when compared to the results that were obtained before they got infected (Table 4). In addition, the running velocities at VT (_V_VT) and RC (_V_RC) were not significantly different post-COVID-19 recovery while the velocity at VO2max (_V_VO2_max_) was significantly lower [t_(20)_ = 2.34, p < 0.05, d = 0.41 (small effect)] (Table 5). Lastly, maximal heart rate (HR_Max_) values remained unchanged while HR values at VT (HR_VT_) [t_(20)_ = −2.79, p < 0.01, d = 0.55 (medium effect)] and RC (HR_RC_)[t_(20)_ = −3.72, p < 0.01, d = 0.52 (medium effect)] were significantly higher post—COVID-19 recovery compared to pre- COVID infection (Table 6).

**Table 3:** Anthropometric and body composition characteristics pre- and pos- COVID-19.

	n	Pre-COVID-19 Infection	Post-COVID-19 Recovery
		Mean ± SD	Mean ± SD
Height (cm)	21	178.21 ± 5.44	
Weight (kg)	21	74.12 ± 5.21	74.20 ± 5.04
Body fat (%)	21	11.40 ± 2.43	11.24 ± 2.28

**Table 4:** Maximal oxygen uptake (VO_2max_), oxygen uptake at ventilatory threshold (VO_2VT)_, and respiratory compensation point (VO_2RC)_ and running time on the treadmill (RT).

	N	Pre-COVID-19 Infection	Post-COVID-19 Recovery
		Mean ± SD	Mean ± SD
VO_2max_ (ml/kg/min)	21	57.35 ± 4.55	54.34 ± 5.24**
VO_2VT_ (ml/kg/min)	21	37.12 ± 6.40	36.89 ± 5.18
VO_2RC_ (ml/kg/min)	21	49.80 ± 6.10	47.47 ± 5.73*
RT (min)	21	17.54 ± 2.21	16.56 ± 2.05**

*p < 0.05, **p < 0.01.

**Table 5:** Running velocities at VO_2max_ (_V_VO_2max_), VT (_V_VT) and RC (_V_RC).

	n	Pre-COVID-19 Infection	Post-COVID-19 Recovery
		Mean ± SD	Mean ± SD
_V_VO2max (km·h^−1^)	21	17.43 ± 1.43	16.86 ± 1.35*
_V_VT (km·h^−1^)	21	10.19 ± 1.66	10.19 ± 1.40
_V_RC (km·h^−1^)	21	13.90 ± 1.73	13.90 ± 1.61

*p < 0.05, **p < 0.01.

**Table 6:** Maximal heart rate (HR_MAX_) and heart rate at VT (HR_VT_), and RC (HR_RC_).

	n	Pre-COVID-19 Infection	Post-COVID-19 Recovery
		Mean ± SD	Mean ± SD
HR_MAX_ (beats·min^−1^)	21	190.14 ± 7.93	191.48 ± 7.67
HR_VT_ (beats·min^−1^)	21	149.90 ± 16.62	158.14 ± 12.97**
HR_RC_ (beats·min^−1^)	21	174.52 ± 9.32	179.57 ± 10.07**

*p < 0.05, **p < 0.01.

## Discussion

To the best of our knowledge, this is the first study to examine the aerobic capacity of professional soccer players pre- and post- COVID-19 infection. Considering that the average intensity in a 90-min soccer game is close to that of the lactate threshold^25^, it is evident that aerobic capacity in soccer is not only dependent on VO2max but also on lactate thresholds and the associated running velocities^26^. Research affirms that enhancement of aerobic capacity leads to improved soccer performance, as it increases the total distance covered by the players, the level of work intensity^38^ as well as the number of sprints during competitive games^27^. This study demonstrated a significant reduction in VO_2max_ and RT on the treadmill about 60 days post COVID-19 recovery. Furthermore, the VO_2RC_ was significantly lower while the VO_2VT_ was reduced but not significantly post COVID infection. Studies highlight the importance of using thresholds to indicate and monitor the improvements in aerobic performance indices during each period of a typical soccer season^28^. More specifically, research indicated that 8-week preseason training caused significant increases in the RC point and VT^28^. Furthermore, research indicated that RC point is inversely correlated with hypoxic ventilatory response and that 40 to 50% of the variance of RC is accounted for by hypoxic ventilatory response and delta slope (rate of lactic acid increase during exercise)^23^. The reduction in the RC point in this study may indicate an increased hypoxic ventilatory response which could be compensated by the cardiovascular system in soccer players. While "silent" hypoxemia has been reported in healthy individuals infected with COVID-19^29^, it is unclear if it persists after recovery in athletes with mild or moderate manifestations. Additionally, prior to the RC point, the cardiorespiratory challenge was not associated with the expiration of the amount of CO_2_, and the need for oxygen was met primarily through the increase in tidal volume and not the respiratory rate^30^. While that is true for VT, the RC point is associated with hyperventilation, that is, the loss of linearity in a plot between VE and V CO_2_. In this study, the earlier occurrence of the RC point, which is associated with hyperventilation due to the failure of bicarbonate buffering and the consecutive fall in blood pH^31^, should be taken into consideration as the significant reductions in aerobic capacity infer a decline in physical performance during the competitive games of the players. Concurrently, these reductions cannot be solely attributed to the detraining period as the athletes followed a 2-week re-training program, a 10-days specific adaptation program, and a 20-days game adaptation program following their recovery (Table 1). Of note is that the players ceased training for only two weeks. While some studies suggest a decline in aerobic capacity even after a short detraining period^13^, others suggest that short-term detraining after a competitive season improved levels of strength and cardiorespiratory fitness in soccer players^16^. Therefore, our findings may raise essential concerns regarding the players' preparedness as we demonstrate that the players have reduced aerobic capacity even 60 days post COVID-19 recovery.

Despite the significant reductions in VO_2max_, RT, andVO_2RC_, the vVT and vRC remained the same. With regards to the velocities, the only significant reduction was indicated on _V_VO_2max_. Research demonstrated that running speeds at maximum lactate steady state are directly related to the ability to use oxygen and, subsequently, to enhance metabolite removal^32^. Additionally, the _V_VO_2max_ in soccer has positively correlated with the distance covered and the running intensity of professional players^33^. Even though _V_VO2max was significantly lower (from 17.43 to 16.86 km/h) following COVID-19 recovery, similar running velocities were reported by others after the preseason training^27^.

With respect to heart rate changes, this study demonstrated significantly higher heart rates at both VT and RC while HR_MAX_ was increased but not significantly following COVID-19 recovery. Considering the reduction of RC point, it could be assumed that increased HR_RC_ and HR_VT_ may be associated with an increased cardiovascular response due to hypoxemia^29^. While increases in submaximal and maximal heart rates have been associated with detraining^34^, the mechanisms involved in the elevation of HR_RC_ and HR_VT_ in our study need further investigation. To date, there are no published studies on the assessment of maximal aerobic capacity using direct measurement of oxygen uptake in athletes post COVID-19. A study of SARS survivors six months after discharge indicated that 75% of the survivors had abnormal tests, out of which 43% had reduced work due to deconditioning, 19% had cardiovascular limitations, and 6% had pulmonary limitations^35^. Another study reported that survivors of ALI (acute lung injury) experience frequent and often distressing pulmonary and psychological symptoms long after their lung injury^36^.

To sum up, all the aforementioned findings indicate that post COVID-19 soccer players may not reach full recovery at two months; therefore, a more detailed evaluation should be conducted before they return to official games. Our results should alert practitioners, fitness coaches, and soccer players of the risk of longer-duration silent symptoms even in athletes that experience mild to moderate manifestations. Practitioners need to be able to adjust the training intensities to gradually enhance the fitness levels of the infected players. Consideration should be given not only to the VO_2max_ and RT on the treadmill but also to other performance indices such as the VT, RC, HR and running velocities at those thresholds. Furthermore, it is suggested that maximal cardiorespiratory testing should be utilized to assess the aerobic capacity of the players before the official games. Since there is the possibility that athletes who contracted COVID-19 have late cardiorespiratory complications, the cardiorespiratory testing can help differentiate low conditioning from cardiorespiratory inefficiency.

## Conclusions

This study demonstrated that a successful return to play necessitates the development of more specific protocols for the safe return of athletes that were infected even if they experienced mild to moderate manifestations. Ideally, those protocols should be determined on a case-by-case basis considering the severity of COVID-19, period of inactivity, and pre-infection fitness level. In addition, consideration should be given to the psychological status^7^, playing position^37^, age, and division in which each player participates, as all the above will contribute to the safe and successful return to training and competition. Lastly, further investigation is needed using GPS technology to evaluate the effect of COVID-19 infection on physical match performance.
